# Supplementary material for: KoVariome: Korean National Standard Reference Variome database of whole genomes with comprehensive SNV, indel, CNV, and SV analyses
Source: Sci Rep. 2018 Apr 4;8:5677. doi: 10.1038/s41598-018-23837-x (PMC5885007; doi:10.1038/s41598-018-23837-x)
Supplement: Supplementary file 1 — Supplementary Figures [file 41598_2018_23837_MOESM1_ESM.docx]

**KoVariome: Korean National Standard Reference Variome database of whole genomes with comprehensive SNV, indel, CNV, and SV analyses**

Jungeun Kim^1†^, Jessica A. Weber^2†^, Sungwoong Jho^1†^, Jinho Jang^3,4^, JeHoon Jun^1, 5^, Yun Sung Cho^5^, Hak-Min Kim^3,4^, Hyunho Kim^5^, Yumi Kim^5^, OkSung Chung^1,5^, Chang Geun Kim^6^, HyeJin Lee^1^, Byung Chul Kim^7^, Kyudong Han^8^, InSong Koh^9^, Kyun Shik Chae^6^, Semin Lee^3,4^, Jeremy S. Edwards^10,*^, and Jong Bhak^1,3,4,5,*^

^1^ Personal Genomics Institute, Genome Research Foundation, Cheongju 28190, Republic of Korea

^2^ Department of Biology, University of New Mexico, Albuquerque, NM 87131, USA.

^3^ Department of Biomedical Engineering, School of Life Sciences, Ulsan National Institute of Science and Technology (UNIST), Ulsan 44919, Republic of Korea

^4^ The Genomics Institute, Ulsan National Institute of Science and Technology (UNIST), Ulsan 44919, Republic of Korea

^5^ Geromics, Ulsan 44919, Republic of Korea.

^6^ National Standard Reference Center, Korea Research Institute of Standards and Science, Daejeon 34113, Republic of Korea.

^7^ Clinomics, Ulsan 44919, Republic of Korea.

^8^ Department of Nanobiomedical Science & BK21 PLUS NBM Global Research Center for Regenerative Medicine, Dankook University, Cheonan 31116, Republic of Korea.

^9^ Department of Physiology, College of Medicine, Hanyang University, Seoul 04763, Republic of Korea.

^10^ Chemistry and Chemical Biology, UNM Comprehensive Cancer Center, University of New Mexico, Albuquerque, NM 87131, USA.

†Contributed equally

*Corresponding authors

E-mail: jsedwards@salud.unm.edu (JE), jongbhak@genomics.org (JB).

**Supplemental Figures**


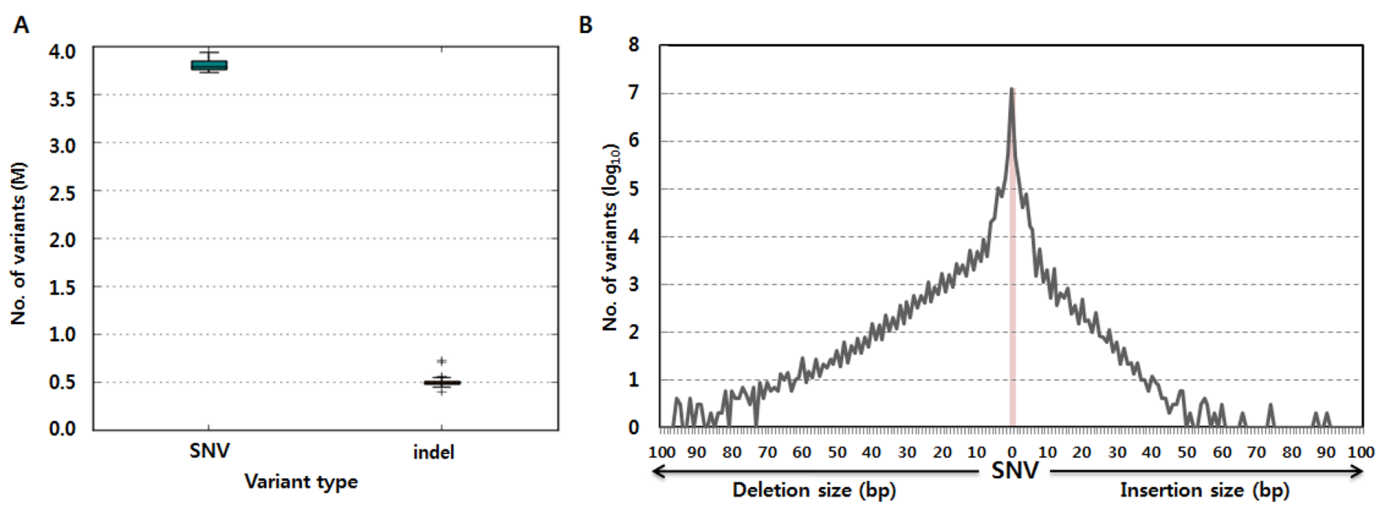


**Figure S1. Distribution of SNP and short indels in 50 Korean individuals**

A. Number of variants present in the Korean individuals. See Table S2 for KoVariome statistics. B. Number of variants according to the variome length.


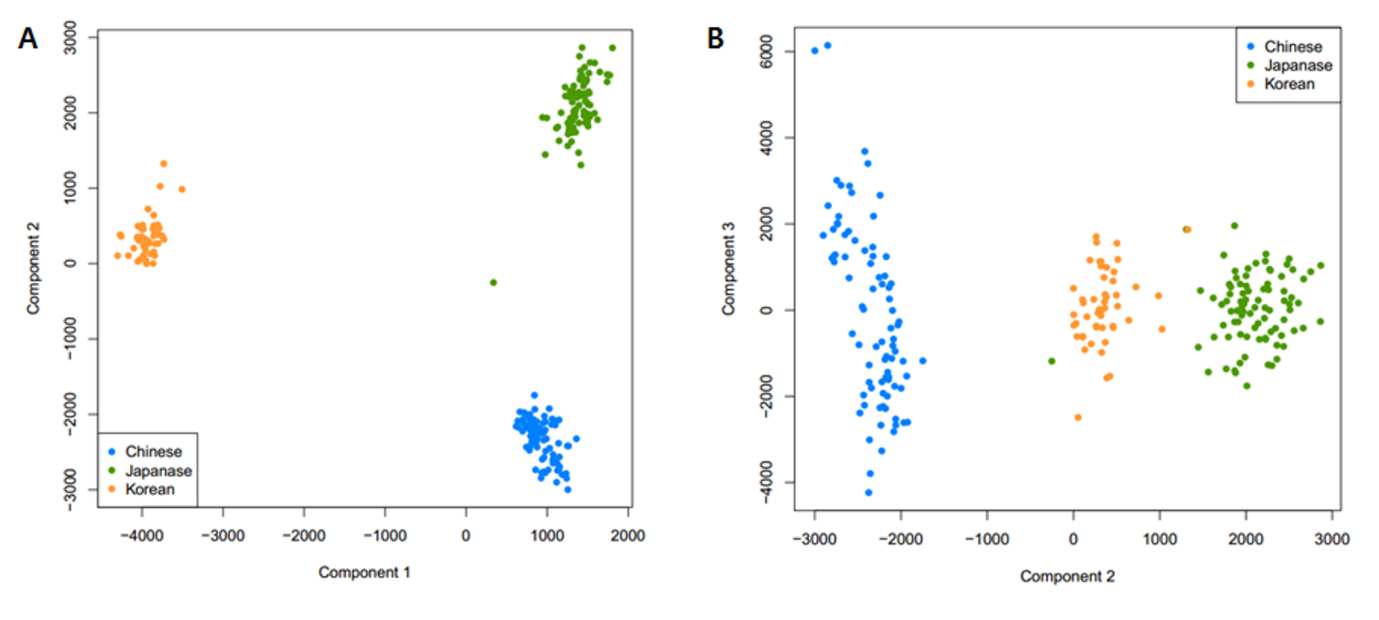


Figure S2. MDS analysis among Korean, Chinese, and Japanese individuals

Genotype data for 84 Chinese and 86 Japanese individuals were merged with KoVariome. Five dimensional components were calculated to identify population stratification between these three ethnic groups.


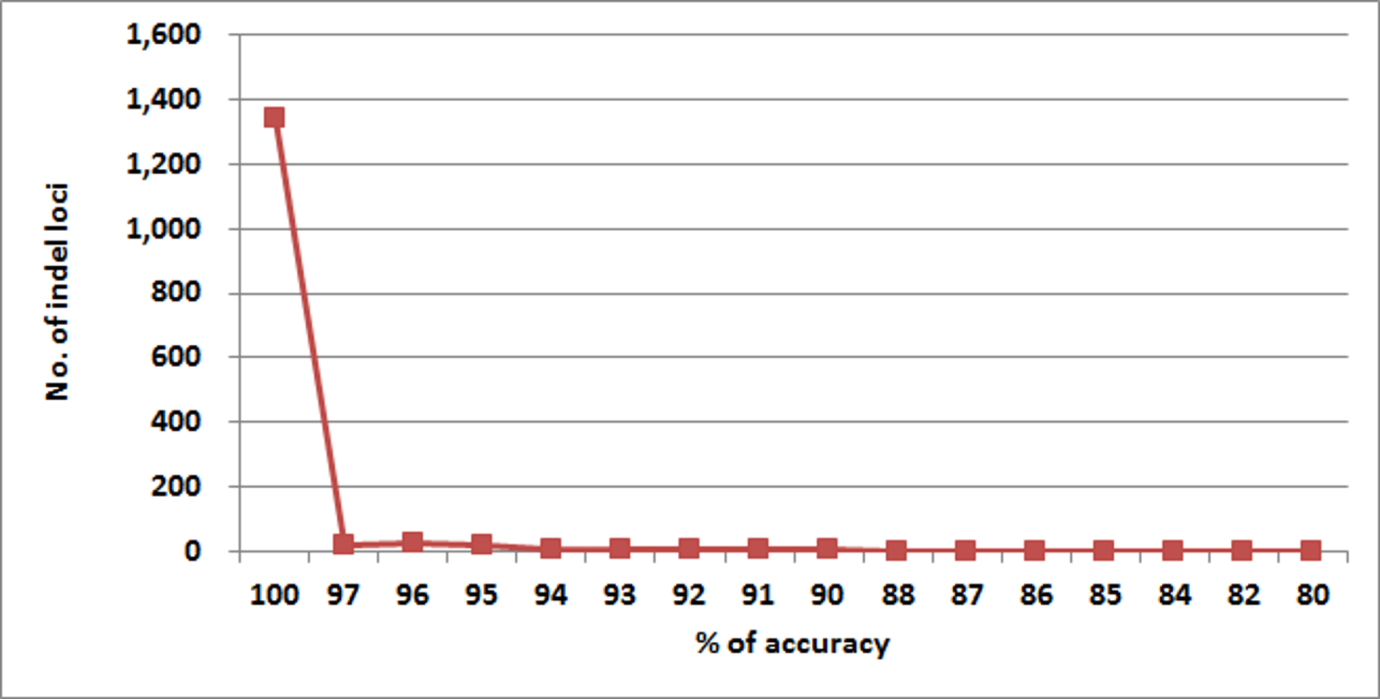


Figure S3. Accuracy by indel loci.

1,343 (91.11%) of KoVariome indels showed perfect genotype concordance with Axiom^TM^ Genome-ASI 1 Array data and 1,446 (98.10%) indels had an accuracy higher than 90%


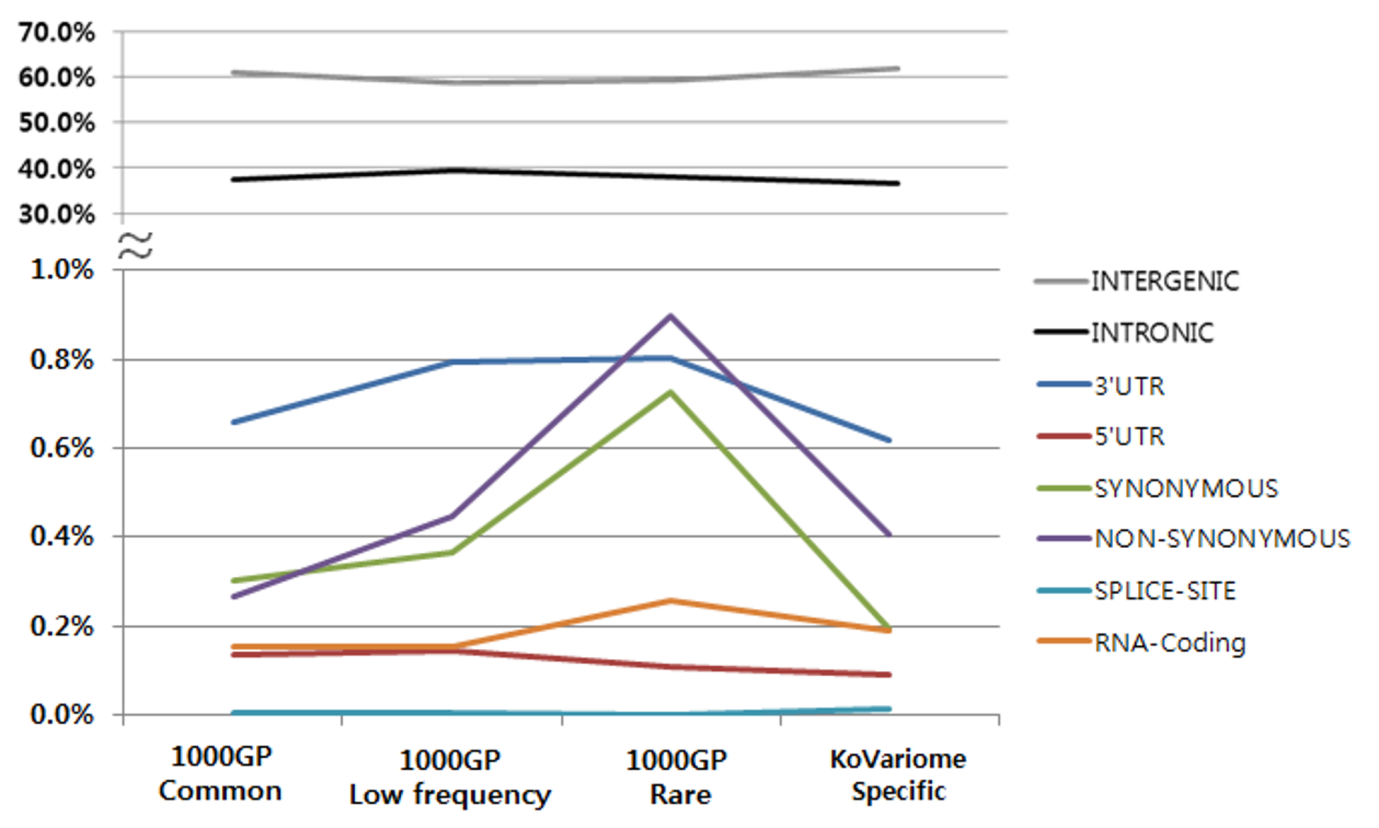


Figure S4. The frequency of KoVariome according to genomic coordinates

The classification of variants observed in 1000GP was also categorized with minor allele frequencies (MAF); ‘1000GP common’ (MAF >= 5% in all continental regions), ‘1000GP low frequency’ (MAF >= 0.1% at any continentals), and ‘1000GP rare’ (MAF <0.01% in all continental regions). SNVs not observed in 1000GP were classified as KPGP specific.


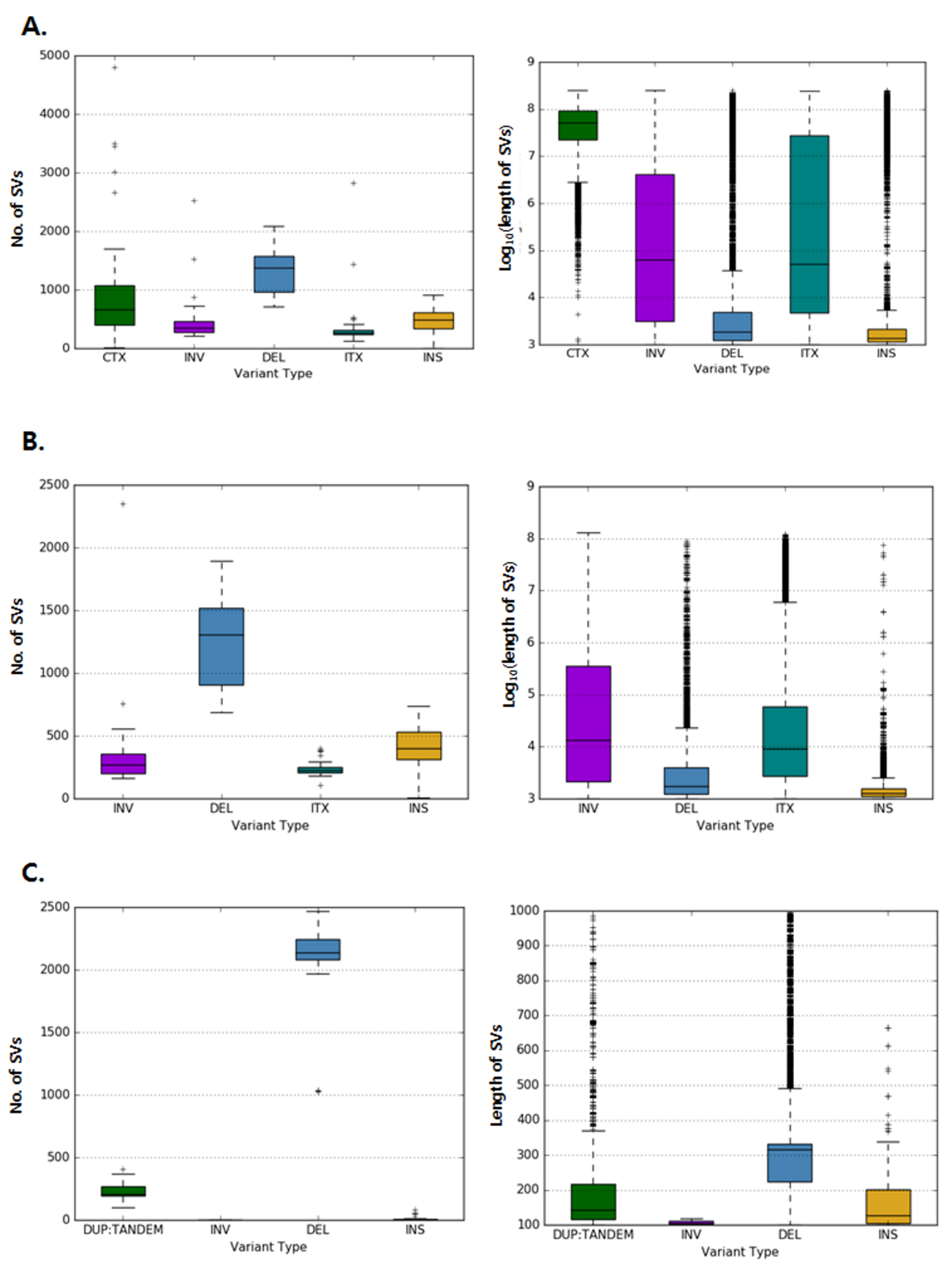


**Figure S5. Comparison of structural variation (SV) statistics**

Abbreviation: inter-chromosomal translocations (CTX), inversions (INV), deletions (DEL), intra-chromosomal translocation (ITX), insertions (INS), duplications (DUP). Box plot represents the number (left) and the length (right) of SVs in the individual genome. A. raw data of SVs predicted by BreakDancer, B. BreakDancer SVs after filtration of spurious SVs, C. SVs predicted by Pindel. The x-axis represents SV types and the y-axis represents the number of SVs (left) and the length of SVs (right). The lower and upper hinges of the boxes correspond to the 25^th^ and 75^th^ percentiles and the whiskers represent the 1.5x inter-quartile range (IQR) extending from the hinges. ‘+’ represents outliers. We didn’t filter the spurious SNVs for CTX because of ambiguous break points between chromosomes. Average Length of the SVs predicted by BreakDancer (A) was significantly longer than those predicted by Pindel (C). We re-distributed SVs after filtering out the SVs containing un-assembled genome of hg19 (B). It shows high portion of large SVs were removed, as spurious SVs, and average length of SVs also lower than raw data. On the other hands, SVs predicted by Pindel (length < 1kbp) were not contained spurious SVs in reference sequences.


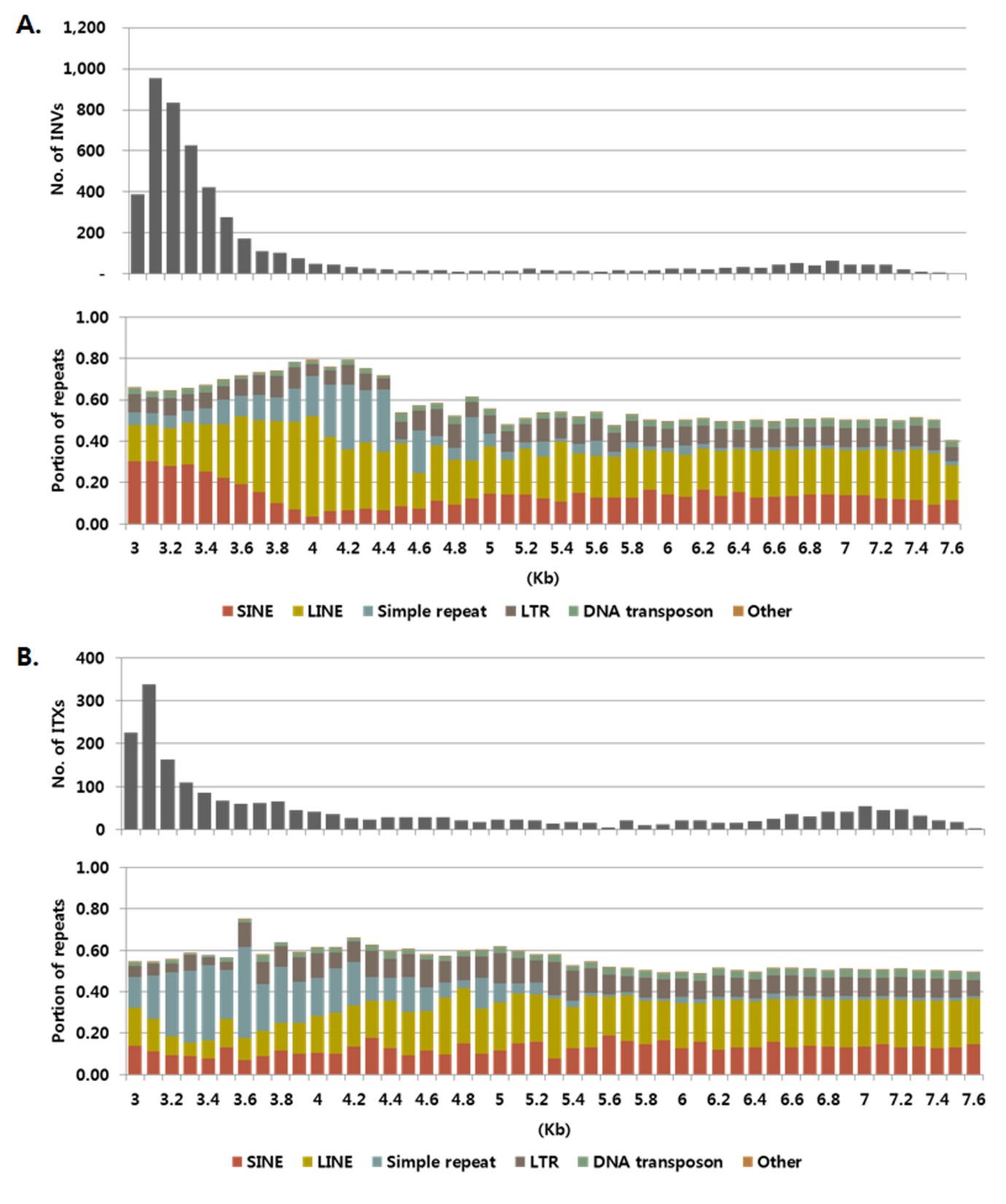


**Figure S6. Length and repeat distribution in inversion and intra-chromosomal translocation**

The graphs represent distributions of SVs (upper) and portion of repetitive sequences at a specific length range. A. inversions (INV), B. intra-chromosomal translocation (ITX) The x-axis represents the SV length of log_10_ scale.

**
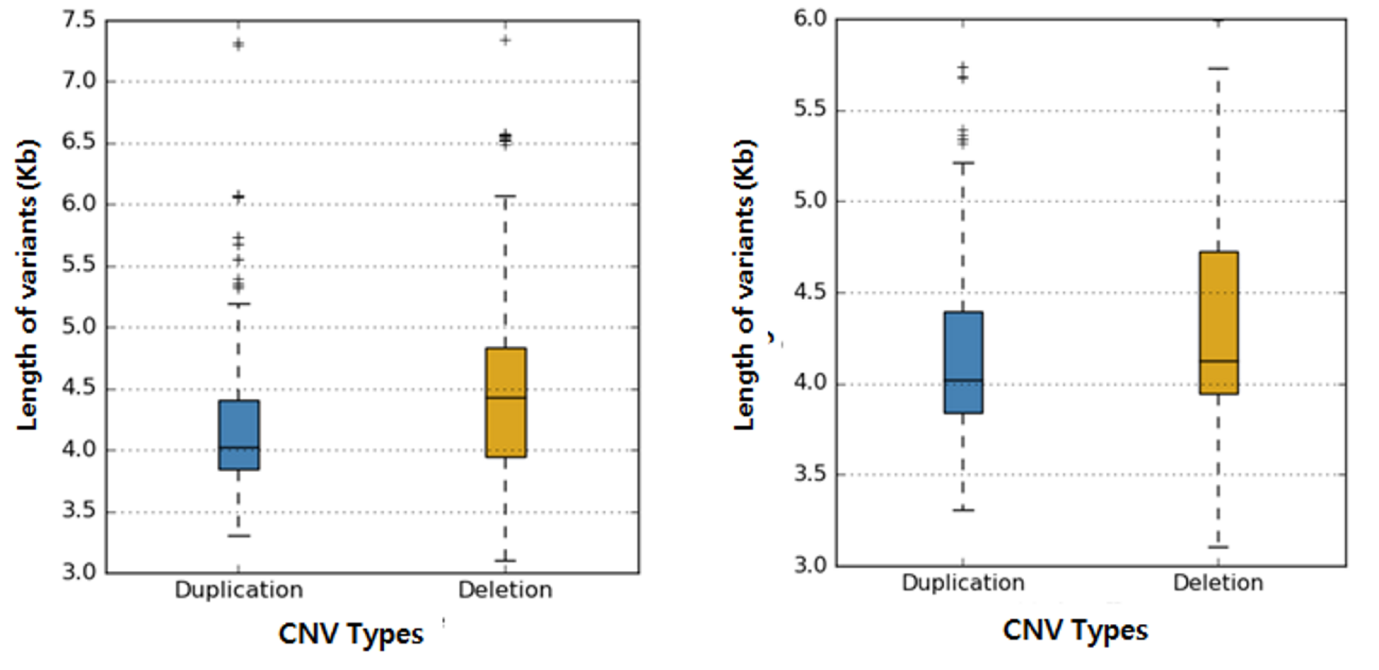
Figure S7. Length distribution of CNVs**

CNVs were predicted by FREEC program. Length distribution of raw data (left) and filtered CNVs in the un-assembled genomic regions (right).
